# Identification and characterization of disease causing genetic variant by conventional genotyping and whole genome sequencing in familial tooth agenesis

**DOI:** 10.1186/1755-8166-7-S1-P26

**Published:** 2014-01-21

**Authors:** Tanmoy Sarkar, Rajesh Bansal, Parimal Das

**Affiliations:** 1 Centre for Genetic Disorders, Faculty of Science, Banaras Hindu University, Varanasi- 221 005, India; 2 Faculty of Dental Sciences, Institute of Medical Sciences, Banaras Hindu University, Varanasi- 221 005, India

## Background

Congenital tooth agenesis (CTA), a type of craniofacial disorders affects approximately 20% (including 3^rd^ molar or Wisdom teeth) and 2-10% (excluding 3^rd^ molar) of the world population. Five major candidate genes known to be associated with syndromic and non-syndromic CTA, these are PAX9, MSX1, AXIN2, EDA and WNT10A. The present investigation was undertaken to identify and characterize disease causing genetic variant by conventional genotyping and whole genome sequencing in familial tooth agenesis

## Material and methods

We have identified two Indian families (DEN11 and DEN12) segregating two distinct types of non-syndromic tooth agenesis with autosomal dominant (DEN11) and apparently indistinguishable either autosomal or X-linked dominant pattern of inheritance (DEN12) family. Eight affected and four unaffected members from DEN11 and four affected and two unaffected members from DEN12 family were enrolled for the present study to identify causative genetic defect associated with the disease. While direct DNA sequencing and microsatellite based genotyping were carried out to screen all but EDA genes in affected (II-5,-7, III-1,-7,-9, IV-2,-3,-4) and unaffected (III-2,-3,-5, IV-1) family members of DEN11, whole genome sequencing was carried out for two affected (III-2 and IV-4) family members from DEN12 along with four unaffected unrelated controls using Illumina 2500 Next Generation Sequencing platform for identification and subsequent characterization of causative variant/s.

## Results and conclusions

In the microsatellite and SNP based haplotype analysis we identified a haplotype block of a ~9.64 Mb region containing PAX9 gene located between D14S70 and D14S288 markers associated with CTA in DEN11. However, absence of any DNA sequence variant within the exons and exon-intron boundaries of the linked PAX9 was found and this indicates the involvement of other pathogenic mechanism. In the whole exome sequencing we identified 86 nonsynonymous novel nucleotide variations distributed among 84 different genes across the nuclear genome of two affected members of DEN12 subjected for investigation. Using bioinformatics, among those variations a specific variation in Ectodysplasin-A (EDA), c.956G>T transversion leading to p.Ser319Ile, at the Tnf homology domain, was considered as a potential pathogenic variation. This variation was not observed in any unaffected family member and in 100 unrelated control chromosomes as assayed through Sanger sequencing and/or RFLP. In silico analysis using SwisSPdb Viewer V4.1.0 reveals that this change destroys an H-bond between p.319 Ser and p.332 Cys establishing this as a plausible pathogenic variation for tooth agenesis in DEN12 family.

